# CRIBAR: a fast and flexible sgRNA design tool for CRISPR imaging

**DOI:** 10.1093/bioadv/vbaf022

**Published:** 2025-02-12

**Authors:** Xiaoli Chen, Md Mahfuzur Rahaman, Ardalan Naseri, Shaojie Zhang

**Affiliations:** Department of Computer Science, University of Central Florida, Orlando, FL 32816, United States; Department of Computer Science, University of Central Florida, Orlando, FL 32816, United States; Department of Computer Science, University of Central Florida, Orlando, FL 32816, United States; Department of Computer Science, University of Central Florida, Orlando, FL 32816, United States

## Abstract

**Motivation:**

CRISPR imaging enables the real-time tracking of nucleic acids. Using guide RNAs (gRNAs) to direct fluorescent tags to target regions allows for precise nucleic acid monitoring via microscopy. The design of gRNAs largely affects the efficacy of CRISPR imaging. Currently, available gRNA design tools are developed primarily for gene editing, often producing individual gRNAs that target genes or regulatory elements.

**Results:**

In this study, we introduce CRIBAR, a computational tool developed to systematically design single-guide RNAs (sgRNAs) for CRISPR imaging applications. CRIBAR first generates sgRNA sets optimized to maximize the number of on-target binding sites and then evaluates the potential off-target effect. The results of the *in silico* experiment show that CRIBAR enables CRISPR imaging in non-repetitive regions.

**Availability and implementation:**

CRIBAR is available as a software package at https://github.com/ucfcbb/CRIBAR and as a web server at http://genome.ucf.edu/CRIBAR.

## 1 Introduction

Clustered Regularly Interspaced Short Palindromic Repeats (CRISPR) systems have been adapted for various applications, including genome editing, regulation, and imaging (Wang *et al.* 2016). Among these applications, CRISPR imaging enables real-time tracking of genomic elements and provides spatial and temporal information, which is inaccessible through traditional methods, such as Chromosome Conformation Capture (3C)-based techniques. In CRISPR imaging systems, the single-guide RNA (sgRNA) directs the imaging probes, such as fluorescent proteins, to specific genomic loci. One of the major challenges in designing such systems is optimizing the signal-to-noise ratio. To generate a strong signal, CRISPR imaging requires multiple imaging probes in the target region. In a typical CRISPR imaging setup, the use of one sgRNA sequence restricts the system to targeting repetitive regions.

Numerous tools have been developed for CRISPR sgRNA design, all following a common foundational workflow (Alkhnbashi *et al.* 2020). First, candidate sgRNAs are identified and then their on-target efficacy and off-target activity are evaluated. The selection of sgRNAs is focused on avoiding any potential off-targets. CRISPR imaging has distinct requirements for sgRNA design, necessitating clustered binding sites within target regions while allowing for a few off-target loci, provided they do not form clusters comparable to those in the target regions.

Computational tools such as CRISPRbar (Ma *et al.* 2018) and JACKIE (Zhu and Cheng 2022) have been developed to design sgRNA for CRISPR imaging. They output one sgRNA for labeling non-repetitive regions by phase separation-mediated signal amplification. In this study, we introduce CRIBAR, a fast and flexible sgRNA design tool developed to facilitate CRISPR imaging in non-repetitive regions by utilizing a set of sgRNAs.

## 2 Methods

**Figure 1.**
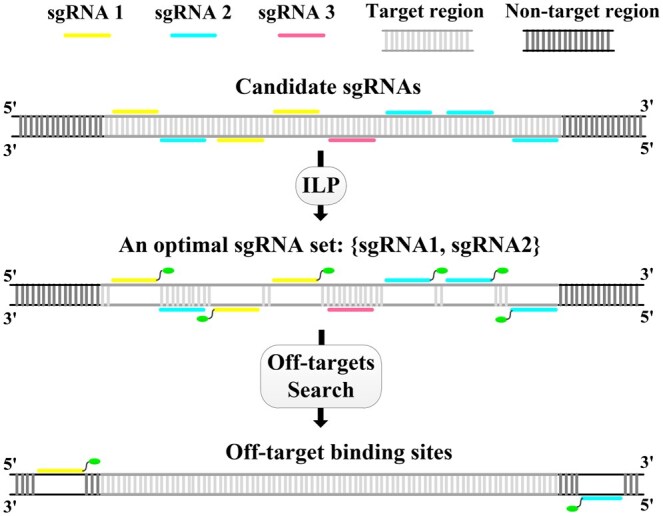
The workflow of CRIBAR is demonstrated by searching for a minimal set of sgRNAs with at least 5 expected binding sites.

The workflow of CRIBAR is illustrated in Fig. 1. For a target genomic region, CRIBAR initiates by exhaustively enumerating the substrings ending with the Protospacer Adjacent Motif (PAM) sequence, considering them as potential candidate sgRNA binding sites. These candidate binding sites are grouped by the sgRNAs. From these sgRNAs, CRIBAR employs Integer Linear Programming (ILP) to select an optimal set that maximizes the on-target efficiency. After this selection, CRIBAR performs an off-target search and evaluates the off-target effect for the chosen sgRNA set. If the calculated off-target score falls below the threshold, CRIBAR outputs the sgRNA set as the final result. Conversely, if the off-target effect exceeds the threshold, CRIBAR generates new optimal sgRNA sets by exploring alternative candidates. This iterative process continues until one of the generated sgRNA sets meets the acceptable off-target score or until all candidate sgRNAs are exhausted. In this way, CRIBAR identifies the most effective sgRNA set for the target genomic region while ensuring an acceptable off-target effect.

### 2.1 Candidate sgRNA enumeration

Given the PAM sequence and the desired length of sgRNAs as input parameters, CRIBAR enumerates all potential sgRNA binding sites within the target region. The candidate sgRNA binding sites are the substrings within the target region that end with the PAM sequence on either of the DNA strands. CRIBAR employs CRISPRitz (Cancellieri *et al.* 2020) for the generation of the candidate sgRNA binding sites. CRISPRitz is implemented using the Aho-Corasick string matching algorithm that exhaustively enumerates the occurrences of the PAM sequence in linear time. When CRISPRitz is unavailable, the alternative built-in brute-force method is executed. Both methods produce the same results. CRISPRitz is set as the primary choice in CRIBAR due to its superior computational efficiency.

Subsequently, CRIBAR retrieves the excluded substrings from a config file and removes all the sgRNAs that contain these substrings. Users can introduce custom substrings by editing this config file or inputting it into our webserver. For instance, adding “TTTT” can help to avoid the potential pre-termination of sgRNA expression under the U6 promoter (Chen *et al.* 2013). Afterward, CRIBAR stores the candidate sgRNA sequences, accompanied by their respective potential binding sites within the target region, organizing them into a hash map. For each sgRNA sequence, the potential binding sites are the substrings containing mismatches less than a user-defined threshold.

### 2.2 Optimal sgRNA set selection

Instead of selecting one sgRNA from candidates, CRIBAR selects a set of sgRNAs. Considering each sgRNA typically has only a few on-target binding sites but may have thousands of off-targets, selecting the optimal sgRNA set by using both on-target and off-target information introduces complexity. To simplify this, CRIBAR initially focuses on selecting sgRNAs based solely on their on-target binding sites and checks their off-targets afterward. CRIBAR leverages ILP to resolve these optimization challenges. ILP formulates optimization problems by maximizing or minimizing an objective while satisfying constraints, with both the objective and constraints being linear.

The design of the sgRNA set focuses on two main goals: minimizing the number of distinct sgRNA sequences and maximizing the number of on-target binding sites. To address this challenge, we first introduce a scoring function that derives from Cutting Frequency Determination (CFD) score (Doench *et al.* 2016), which assesses the binding probability for each binding site by considering the number and positions of mismatches. Generally, more mismatches lead to lower binding probability. Using this scoring function, the sum of expected binding sites is equivalent to the activity score. Then, we approach the sgRNA selection problem through two distinct optimization scenarios. In the first scenario, the objective is to generate an sgRNA set with a specified limit on the number of sgRNA sequences, while maximizing the expected on-target activity score. Conversely, the second scenario aims to generate an sgRNA set with the minimum number of distinct sgRNA sequences, while ensuring a minimum threshold of expected on-target activity score. Both scenarios are formulated as ILP problems, as described in the Supplementary Methods in detail.

### 2.3 Off-target evaluation

After generating an optimal sgRNA set, CRIBAR evaluates its off-target effects. To meet the requirements of CRISPR imaging, CRIBAR assesses off-target effects using the ratio of on-target to off-target activity scores, instead of the absolute number of off-target binding sites. The on-target activity score is determined as discussed in the previous subsection. Similarly, the off-target activity score is calculated as the maximum score across all sliding windows throughout the genome, excluding the target region. The activity ratio is then obtained by dividing the on-target activity score by the off-target activity score. If this ratio is lower than a specified threshold (the default value is 0.2), CRIBAR outputs the sgRNA set. Otherwise, CRIBAR removes the sgRNA with the highest off-target activity score and repeats the selection process until a set of sgRNAs passes the off-target evaluation, or all candidate sgRNAs are exhausted.

### 2.4 Implementation and release of CRIBAR

CRIBAR is implemented as a software package and a web server. The software package is developed in Python, while the web server is built using the Django framework. To generate a set of sgRNAs, the users need to specify the reference genome, target genomic location, sgRNA length, PAM sequence, formulation type, and a constraint value. The constraint value represents the maximum number of distinct sgRNA sequences when the formulation type is set to 1, and the minimum on-target activity score when the formulation type is set to 2. The advanced options include the window size and the activity ratio threshold for off-target effect evaluation. CRIBAR offers two options for off-target search engines: an exhaustive method CRISPRitz (Cancellieri *et al.* 2020) and a heuristic method Bowtie (Langmead *et al.* 2009). Both tools are flexible in the mismatch/indel numbers and the query length. Bowtie employs a heuristics algorithm, which compromised accuracy and sensitivity for enhanced speed. The software package provides both Bowtie and CRISPRitz as options. Our web server uses Bowtie for faster runtime. CRIBAR has been tested with CRISPRitz v2.6.6 and Bowtie v1.2.3.

## 3 Results

### 3.1 Genome-wide accessibility

Empirically, 25 binding sites within a 50 kb window constitute a high-density cluster capable of generating a sufficiently strong signal for CRISPR imaging (Ma *et al.* 2016, 2018, Yang *et al.* 2024). Genomic regions containing such a high-density 50 kb window are considered accessible. For windows of a specified size, accessible region coverage is defined as the ratio of the number of accessible windows to the total number of windows across the entire genome.

**Figure 2.**
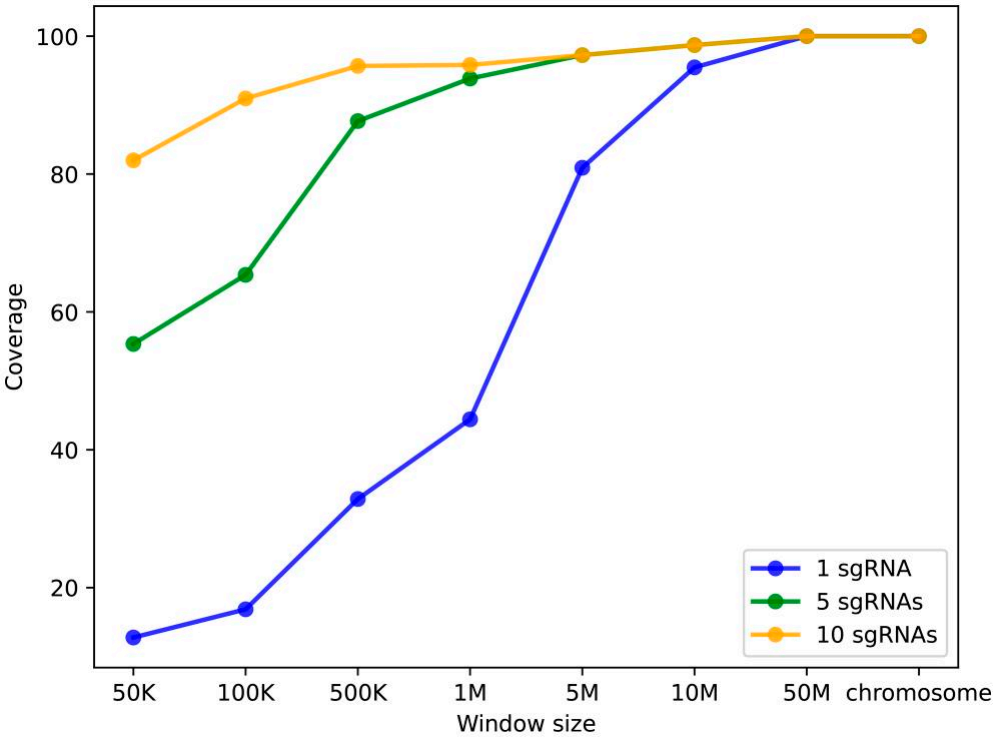
Accessible region coverage of the human genome by using 1, 5, and 10 sgRNAs.

We conducted a genome-wide assessment across the human genome (hg38). The human genome was partitioned into fixed-length regions (50 kb, 100 kb, 500 kb, 1 mbp, 5 mbp, 10 mbp, 50 mbp, and entire chromosomes). The accessible region coverage was evaluated using sets of 1, 5, or 10 distinct sgRNA sequences. The evaluation results for the first formulation are summarized in Fig. 2. For 50 kb windows, 12% were accessible by using 1 sgRNA, while 82% became accessible with a set of 10 sgRNAs. For 1 mbp windows, 40% were accessible by using 1 sgRNA, and this percentage increased to 95% when using a set of 10 sgRNAs. This result suggests that for users aiming to employ CRISPR imaging for tracking the three-dimensional structures of the genome at a resolution of 1 mbp, a set of 10 sgRNAs should generally suffice. The distribution of the high-density 50 kb window is shown in Supplementary Fig. S1. By using a set of 10 sgRNAs instead of 1 sgRNA, CRIBAR improved the coverage of accessible regions across the human genome from 12.8% to 82%.

For the second formulation, we set a minimum of 25 on-target binding sites as the constraint and used CRIBAR to search for the smallest sgRNA sets. The minimum sgRNA number distribution for all the 50 kb windows across the human genome is shown in the heatmap in Supplementary Fig. S2A. A cumulative plot of the minimum sgRNA numbers is presented in Supplementary Fig. S2B. By using 25 sgRNAs, CRIBAR generated sgRNA sets for 87.6% of the 50 kb windows. As indicated in gray and white in Supplementary Fig. S2A, 8.8% of the windows lack sequences, and 3.6% failed the off-target check for all candidate sgRNA sets.

### 3.2 Case study

In this section, we demonstrate the utility of CRIBAR with an experiment targeting the genomic region chr20:20 000 000–20 050 000 in the human genome, performed using both formulations. For both formulations, the constraint value is set to 10, the PAM sequence is set to NGG, and the maximum mismatches allowed when searching for binding sites are set to 3. Furthermore, the off-target ratio threshold is 0.2, the minimum gap between any two sgRNA binding sites is 30 bp, and excluded substrings include “AAAA” and “TTTT.” The CFD score is used for the scoring. CRIBAR completed the design for both formulations within 2 min on a Linux machine equipped with an Intel i7-8700 CPU. The majority of the runtime was consumed by the ILP solver, as it generally requires multiple iterations to achieve an optimal result. It is also worth noting that each run of CRIBAR may produce slightly different outputs due to the randomness of the ILP solver. The results of the experiment are as follows.

**Figure 3.**
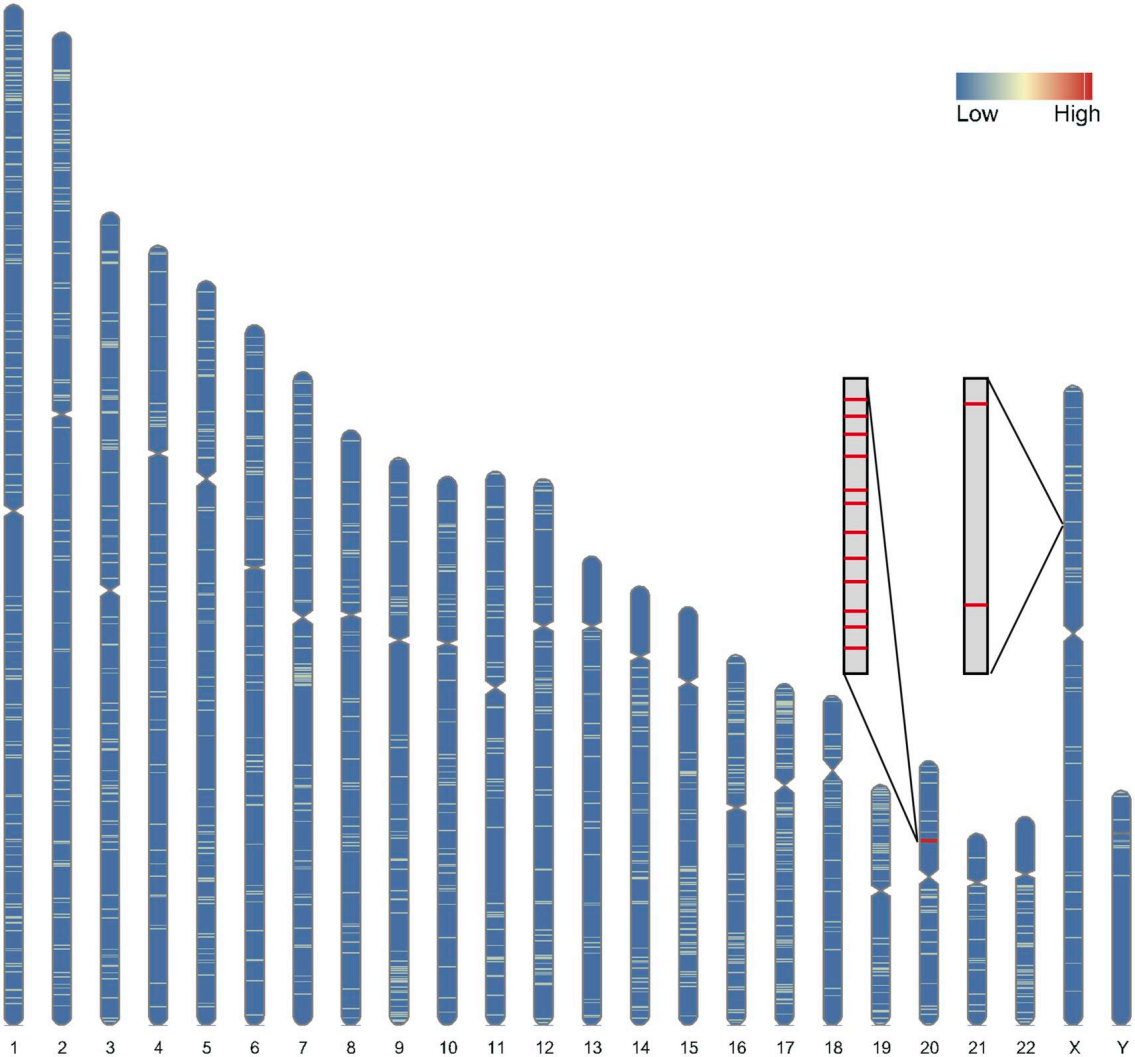
The results for the second formulation in the case study. The heatmap illustrates the number of binding sites across the human genome for the selected sgRNA set. Zoom-in views are provided for both the target region and a representative non-target region. Within the target region, 12 binding sites are identified, whereas the non-target region contains 2 binding sites.

In the experiment using the second formulation, CRIBAR was used to identify a minimal sgRNA set with an on-target activity score of at least 10. This resulted in a set of 6 distinct sgRNAs, collectively generating 12 potential binding sites within the target region and achieving a total on-target activity score of 10.03. A heatmap of the binding sites for the selected sgRNA sets across all 50 kb windows in the human genome is shown in Fig. 3, where the target region exhibits a significantly higher activity score compared to all other windows. Using the first formulation, CRIBAR was used to design a sgRNAs set with at most 10 distinct sgRNAs that maximize the expected binding site. The output included 10 distinct sgRNAs, generating 25 expected binding sites within the target region and achieving a total on-target activity score of 17.5.

## 4 Conclusion and discussion

Here, we introduced a novel sgRNA design tool for CRISPR imaging applications, named CRIBAR. It designs a set of sgRNAs to address the limitation of functioning only within repetitive regions. Using more sgRNA sequences increases the cost and complexity of the CRISPR imaging experiment. CRIBAR employs ILP to generate the optimal set of sgRNAs based on the on-target activity score, followed by off-target effect assessment. If the assessment indicates an unsatisfactory off-target effect, CRIBAR removes the candidate sgRNA with the most severe off-target effect and repeats the ILP. In rare cases, this heuristic strategy may lead to sub-optimal results or even failure to generate any results, as the excluded sgRNA might belong to the optimal sgRNA set. The reason for using this iterative two-step heuristic strategy is that non-target regions are vast and may contain thousands of potential binding sites for each sgRNA, making it impractical to include them in the ILP formulation in a single step.

As shown in Section 3, CRIBAR is capable of accessing 95% of the human genome using a set of 10 sgRNAs. Potential factors contributing to the failure to generate sgRNAs for the remaining 5% include (i) an insufficient number of sub-sequences in the target region containing the PAM sequence; (ii) significant similarity between the primary sequence of the target region and non-target regions; and (iii) the heuristic strategy in CRIBAR may have discarded an optimal sgRNA candidate. In addition to the potential for sub-optimal results or failure to generate any results, it is also noted that each execution of CRIBAR may yield different outcomes due to the stochastic nature of the ILP solver.

One limitation of the current version of CRIBAR comes from the scoring function. The scoring function is specifically designed for the sgRNAs of 23 bp. If the desired sgRNA length differs from 23 bp, CRIBAR assigns uniform activity scores to all binding sites. Additionally, although this scoring function accounts for the impact of mismatches at potential binding sites, it does not consider other factors, such as chromatin structure, that may influence sgRNA binding specificity. To address this limitation, CRIBAR can be extended to integrate alternative scoring functions in future studies.

## Supplementary Material

CRIBAR_Supplementary_Materials.pdf

## References

[vbaf022-B1] Alkhnbashi OS , Meier T, Mitrofanov A et al CRISPR-Cas bioinformatics. Methods 2020;172:3–11. 31326596 10.1016/j.ymeth.2019.07.013

[vbaf022-B2] Cancellieri S , Canver MC, Bombieri N et al CRISPRitz: rapid, high-throughput and variant-aware in silico off-target site identification for CRISPR genome editing. Bioinformatics 2020;36:2001–8. 31764961 10.1093/bioinformatics/btz867 PMC7141852

[vbaf022-B3] Chen B , Gilbert LA, Cimini BA et al Dynamic imaging of genomic loci in living human cells by an optimized CRISPR/cas system. Cell 2013;155:1479–91. 24360272 10.1016/j.cell.2013.12.001 PMC3918502

[vbaf022-B4] Doench JG , Fusi N, Sullender M et al Optimized sgRNA design to maximize activity and minimize off-target effects of CRISPR-Cas9. Nat Biotechnol 2016;34:184–91. 26780180 10.1038/nbt.3437 PMC4744125

[vbaf022-B5] Langmead B , Trapnell C, Pop M et al Ultrafast and memory-efficient alignment of short DNA sequences to the human genome. Genome Biol 2009;10:R25. 19261174 10.1186/gb-2009-10-3-r25 PMC2690996

[vbaf022-B6] Ma H , Tu L-C, Naseri A et al Multiplexed labeling of genomic loci with dCas9 and engineered sgRNAs using CRISPRainbow. Nat Biotechnol 2016;34:528–30. 27088723 10.1038/nbt.3526 PMC4864854

[vbaf022-B7] Ma H , Tu L-C, Naseri A et al CRISPR-Sirius: RNA scaffolds for signal amplification in genome imaging. Nat Methods 2018;15:928–31. 30377374 10.1038/s41592-018-0174-0 PMC6252086

[vbaf022-B8] Wang H , La Russa M, Qi LS et al CRISPR/Cas9 in genome editing and beyond. Annu Rev Biochem 2016;85:227–64. 27145843 10.1146/annurev-biochem-060815-014607 PMC13384723

[vbaf022-B9] Yang L-Z , Min Y-H, Liu Y-X et al CRISPR-array-mediated imaging of non-repetitive and multiplex genomic loci in living cells. Nat Methods 2024;21:1646–57. 38965442 10.1038/s41592-024-02333-3

[vbaf022-B10] Zhu JJ , Cheng AW. JACKIE: fast enumeration of genome-wide single- and multicopy CRISPR target sites and their off-target numbers. CRISPR J 2022;5:618–28. 35830604 10.1089/crispr.2022.0042 PMC9527058

