# Supplementary material for: CRIBAR: a fast and flexible sgRNA design tool for CRISPR imaging
Source: Bioinform Adv. 2025 Feb 12;5(1):vbaf022. doi: 10.1093/bioadv/vbaf022 (PMC11846663; doi:10.1093/bioadv/vbaf022)
Supplement: CRIBAR_Supplementary_Materials.pdf [file vbaf022_supplementary_data.pdf]

Supplementary Materials for ‘CRIBAR: a fast and flexible sgRNA design tool for CRISPR imaging’

Xiaoli Chen †, Md Mahfuzur Rahaman †, Ardalan Naseri ‡ and Shaojie Zhang *

Department of Computer Science, University of Central Florida, Orlando, FL 32816, USA
†These authors contributed equally to this work.
‡Present address: McWilliams School of Biomedical Informatics, University of Texas Health Science Center at Houston, Houston, TX 77030, USA
*Corresponding author. E-mail: shzhang@cs.ucf.edu

Supplementary Methods

Integer linear programming formulations

To find the optimal sgRNA sets from the on-target binding sites, we define a scoring function based on a scoring algorithm that derives from Cutting Frequency Determination (CFD) score [1]. Leveraging this algorithm, we define the on-target activity score for a set of sgRNAs as the expected number of their binding sites within the target region. Recent works [2] attempted to interpret the features that affect sgRNA specificity and efficiency by using machine learning. These methods typically train rank-based regression models on normalized sgRNA abundance, which may not represent the absolute activity of the sgRNAs and are not suitable for calculating total activities and activity ratios.

Formulation 1:

$$Obj:\ max\sum_{i=1}^{l}\sum_{j=1}^{m_i} score_i^j * b_i^j$$

$$Con1:\ s_i = 1 \ \text{or} \ 0$$

$$Con2:\ b_i^j = 1 \ \text{or} \ 0$$

$$Con3:\ b_{i1}^{j1} + b_{i2}^{j2} <= 1 \quad \text{if } b_{i1}^{j1} \text{ conflicts with } b_{i2}^{j2}$$

$$Con4:\ \sum_{k=1}^{m_i} b_j^k <= m_i * s_i$$

$$Con5:\ \sum_{i=1}^{l} s_i <= sgRNA_{threshold}$$

Formulation 2:

$$Obj: min\sum_{i=1}^{l} s_i$$

$$Con1:\ s_i = 1 \ \text{or} \ 0$$

$$Con2:\ b_i^j = 1 \ \text{or} \ 0$$

$$Con3:\ b_{i1}^{j1} + b_{i2}^{j2} <= 1 \quad \text{if } b_{i1}^{j1} \text{ conflicts with } b_{i2}^{j2}$$

$$Con4:\ \sum_{k=1}^{m_i} b_j^k <= m_i * s_i$$

$$Con5:\ \sum_{i=1}^{l}\sum_{j=1}^{m_i} score_i^j * b_i^j >= score_{threshold}$$

By utilizing the scoring function, the potential on-target binding sites in our formulations can be represented by the on-target activity score. For l sgRNA candidates, we denote s_i as the ith sgRNA. s_i is set to 1 if the ith sgRNA is selected, otherwise, it is set to 0. Additionally, we represent the jth binding site of the ith sgRNA as b_i^j and its activity score as $score_i^j$. The ith sgRNA has m_i potential binding sites in total. b_i^j=1 if the corresponding binding site is selected, and b_i^j=0 otherwise. With these notations, we define the Constraints and Objectives in the ILPs for the two optimization scenarios.

The first formulation targets to generate an sgRNA set with a specified limit of sgRNA sequences, while maximizing the expected on-target binding sites. Conversely, the second formulation aims to generate an sgRNA set with the minimum number of distinct sgRNA sequences, while ensuring a minimum number of expected on-target binding sites. In both formulations, the Constraint 1 to 4 remain consistent, whereas the Constraint 5 and the Objective differ. The first constraint (Con1) ensures that each sgRNA can only be selected once. The second constraint (Con2) guarantees that each binding site can only be chosen once. For the third constraint (Con3), the distance between any two binding sites b_{i1}^{j1} and b_{i1}^{j2} must be greater than a predefined threshold (default value is 30 bp). This constraint accounts for sufficient spacing between sgRNA binding sites to accommodate image probes adequately dictates that if any binding sites of an sgRNA are chosen, the sgRNA is considered selected. This constraint transfers the constraint on binding sites to the sgRNAs. The forth constraint (Con4) is to ensure if an sgRNA is not selected, any of its binding sites would not be considered. In formulation 1, the last constraint (Con5) ensures that the number of selected sgRNA is lower than the pre-defined threshold. The objective (obj) is to maximize the on-target activity score. On the other hand, formulation 2 introduces a different constraint (Con5) that ensures the number of expected on-target binding sites exceeds a predefined threshold. The objective (obj) in this case is to minimize the selected sgRNA. CRIBAR leverages GLPK (GNU Linear Programming Kit), to solve these two ILP problems.

Supplementary Figures

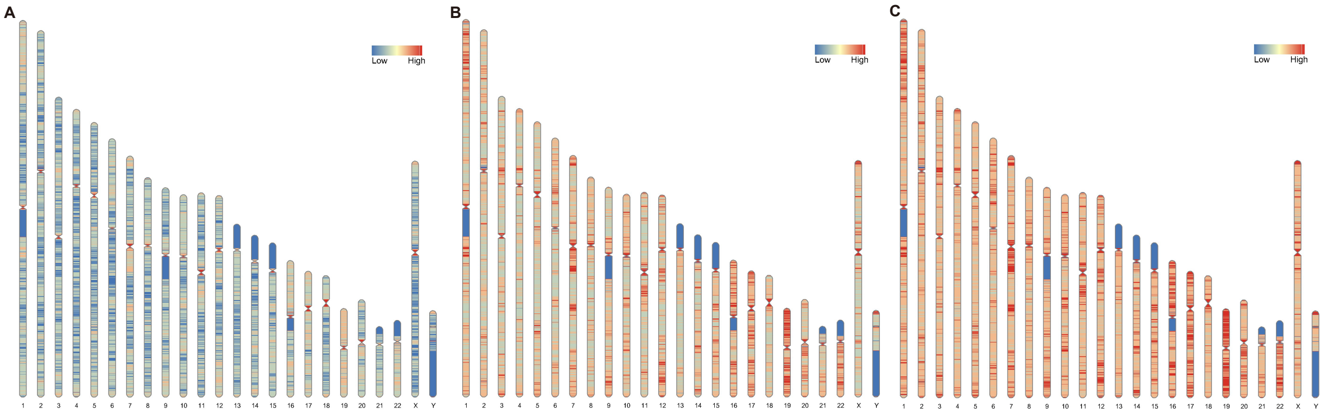

Figure S1: The heatmaps of maximum on-target activity scores of all 50 kbp windows in the human genome by using different numbers of sgRNAs. (**A**) The heatmap for 1 sgRNA. (**B**) The heatmap for 5 sgRNAs. (**C**) The heatmap for 10 sgRNAs.

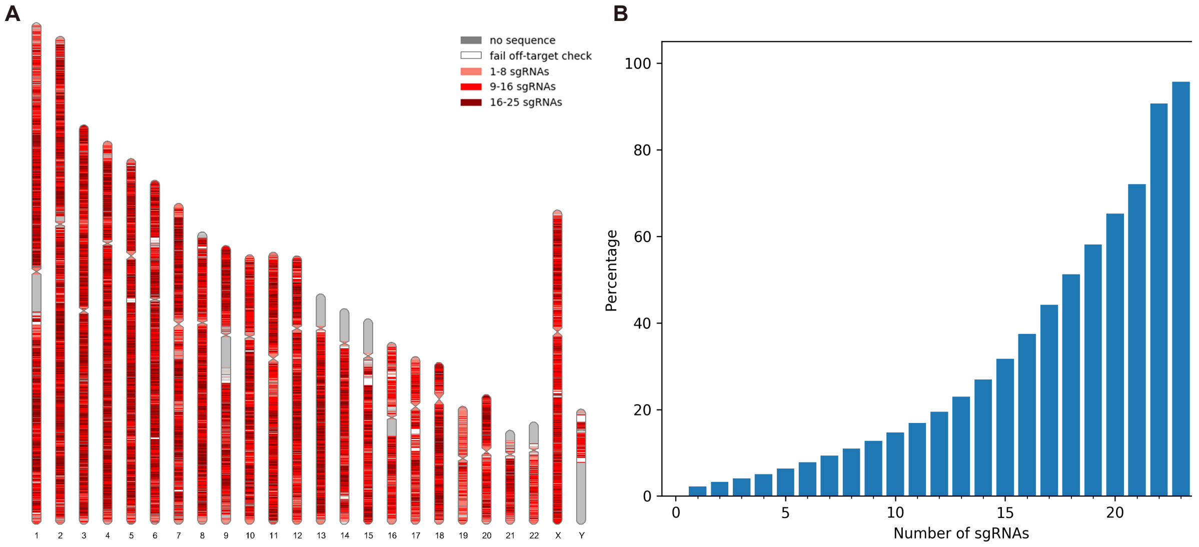

Figure S2: The minimum distinct sgRNAs that CRIBAR used to generate an on-target activity score of 25 for all 50 kbp bins in the human genome. (**A**) The heatmap shows the distribution of the minimum number of sgRNAs across the human genome. (**B**) The cumulative plot shows the statistics distribution of the minimum number of sgRNAs.

References

[1] Doench, J. G., Fusi, N., Sullender, M., Hegde, M., Vaimberg, E. W., Donovan, K. F., Smith, I., Tothova, Z., Wilen, C., Orchard, R., Virgin, H. W., Listgarten, J., and Root, D. E. (2016) Optimized sgRNA design to maximize activity and minimize off-target effects of CRISPR-Cas9. *Nat Biotechnol,* **34**, 184–191.

[2] Chuai, G., Ma, H., Yan, J., Chen, M., Hong, N., Xue, D., Zhou, C., Zhu, C., Chen, K., Duan, B., Gu, F., Qu, S., Huang, D., Wei, J., and Liu, Q. (2018) DeepCRISPR: optimized CRISPR guide RNA design by deep learning. *Genome Biol,* **19**, 80.
